# Simulations on Scintillator Thicknesses for Bone Mineral Density Measurement Based on Dual-Layer Flat-Panel Detectors

**DOI:** 10.3390/diagnostics16182891

**Published:** 2026-09-08

**Authors:** Jongin Kim, Dong Sik Kim, Eunae Lee

**Affiliations:** 1 Division of Semiconductor and Electronics Engineering, Hankuk University of Foreign Studies, Yongin 17035, Gyeonggi, Republic of Korea; hetsx569@hufs.ac.kr (J.K.); dskim@hufs.ac.kr (D.S.K.); 2 Neuroscience Research Institute, Medical Research Center, Seoul National University, Seoul 03080, Republic of Korea

**Keywords:** bone mineral density (BMD), dual-layer flat-panel detector (DFD), dual-energy radiography, multiple linear regression

## Abstract

**Background/Objectives:** A dual-layer flat-panel detector (DFD), in which two flat-panel detectors are stacked vertically, enables single-shot dual-energy imaging without a fan-beam scanning and switching mechanism in conventional dual-energy X-ray absorptiometry (DXA), the clinical standard for bone mineral density (BMD). However, because single-shot BMD measurement systems using DFDs show substantial spectral overlap, their BMD measurement performance is inherently inferior to that of dual-shot systems. In this paper, we optimize the tube voltage, metal filter thickness, and CsI(Tl)-scintillator thickness so that the BMD estimation error of the single-shot method is similar to that of the double-shot method. Here, we also optimize the dual-shot approach to serve as a meaningful reference in the comparison. **Methods:** BMD measurement simulations were performed using a polynomial estimator based on second-order polynomial fitting of dual-energy logarithmic intensities. Performance comparison was based on noise sensitivity, quantified by the condition number and mean square error under a multiplicative noise model, and was further assessed using the equivalent energies and the bone-tissue attenuation ratios. **Results:** The simulation results indicate that, in the dual-shot approach, decreasing the low tube voltage is the most effective strategy for improving BMD measurement performance, whereas in the single-shot approach, reducing the upper scintillator thickness has the largest impact. **Conclusions:** When both approaches are evaluated under their respective optimized configurations based on synthetic simulations, the single-shot approach demonstrates that the BMD estimation error is sufficiently similar to that of the dual-shot approach, supporting its potential as a hardware-efficient alternative for BMD measurement.

## 1. Introduction

Bone mineral density (BMD) measurement is performed using dual-energy X-ray absorptiometry (DXA) systems, which have become the standard tool for osteoporosis diagnosis due to their low radiation dose and proven reliability [1]. In DXA, dual-energy images are acquired by switching tube voltages and metal filters or through photon-counting detectors, and two-dimensional images are reconstructed through a scanning process using a very narrow fan beam and an aligned one-dimensional detector. To obtain accurate images, the fan beam and detector should be translated synchronously [2] during scanning, and additional hardware is required to switch the metal filter according to the tube voltage. This configuration not only increases the system size and cost but also has a fundamental limitation: patient motion between the two exposures and slight mechanical instability can cause misregistration between the dual-energy images.

A dual-shot approach using a flat-panel detector (FD) can acquire two-dimensional images simply, without scanning, by using cone-beam X-rays instead of fan-beam X-rays [3]. However, the FD-based dual-shot approach still relies on two separate X-ray exposures and suffers from the complex switching mechanisms and misregistration issues inherent in conventional DXA systems [4].

To overcome the structural limitations of the dual-shot approach, a single-shot approach based on a dual-layer flat-panel detector (DFD), in which two FDs are stacked vertically, has been proposed. In a DFD, the upper FD primarily absorbs relatively low-energy photons, whereas the lower FD mainly absorbs high-energy photons that have undergone beam hardening [5,6], so that two different energy spectra can be acquired simultaneously with a single X-ray exposure [7]. As a result, without using complex switching mechanisms, existing X-ray systems can be upgraded to a single-shot BMD measurement system at relatively low additional cost by replacing only the detector part with the DFD [8]. Because both images are obtained in a completely simultaneous exposure, image misregistration can be fundamentally eliminated. Lee and Kim [8] demonstrated the feasibility of BMD measurement using a DFD-based single-shot approach for existing DFD prototypes with restricted scintillator thicknesses and intermediate metal filters. They considered an additive noise for the noise sensitivity analysis. By deploying this DFD-based single-shot system, opportunistic BMD screening can be integrated into routine X-ray examinations, where the system provides approximate BMD estimates and flags patients who should undergo standard DXA measurement.

In dual-energy imaging, the performance of BMD measurement depends on the separation between the absorbed spectra [9]. In the single-shot approach based on DFDs, in addition to the nonlinearity and scattering problems caused by the use of FDs, there is a problem of significant spectral overlap between the two absorption spectra obtained from the upper and lower layers. This problem arises because, unlike the dual-shot approach that uses a switching mechanism, in DFDs, the two spectra are generated at the same tube voltage and are separated only by the beam hardening effect in the upper scintillator layer or by an intermediate filter. Because of this substantial spectral overlap, DFD-based BMD measurement systems, although attractive in terms of simple hardware configuration and cost, are at a disadvantage in BMD measurement performance compared with the dual-shot approach.

In this paper, we first address these spectral limitations by comparing the dual-shot and single-shot approaches within a unified simulation framework and exploring the impact of scintillator thickness, together with tube voltage and metal filter settings. In practical BMD measurement systems, nonlinear response and scattering distortion are important issues. However, we neglected these effects and focus on comparing idealized BMD measurement performance, with the aim of providing preliminary information on hardware design. We then conducted simulations to optimize the BMD measurement performance of the DFD-based single-shot approach as close as possible to the dual-shot approach case. Here, the parameter for optimization is the scintillator thickness, based on the tube voltage and metal filter settings. For the simulations, we used SPEKTR 3.0 [10] to synthesize absorbed spectra and conducted multiple linear regression analyses to define a second-order polynomial estimator [4,11]. We then performed noise sensitivity analyses to quantitatively assess and optimize the BMD measurement performance. Here, we calculated the absolute condition numbers and mean square error (MSE) values under multiplicative noise to the inputs. Note that this multiplicative noise can model the fluctuating image intensity from X-ray tube current instability and detector nonlinearity. Furthermore, we introduce equivalent energies and bone-tissue attenuation ratios to analyze the BMD measurement performance from the perspective of spectral and material separation. These metrics provide physical insights that complement the condition number and MSE-based noise sensitivity analysis.

The rest of this paper is organized as follows: In Section 2, we introduce the DFD hardware structure, define the dual-shot and single-shot simulation cases, and present the polynomial estimator based on the multiple linear regression. The absolute condition number and MSE curve are also introduced for the noise sensitivity evaluation. Section 3 presents the simulation results for both approaches, Section 4 discusses the results defining equivalent energies and bone-tissue attenuation ratios, and Section 5 concludes the paper.

## 2. Materials and Methods

In this section, we briefly introduce the DFD and describe the spectrum synthesis for dual-energy imaging used for the simulations. We then define the polynomial estimator for the BMD measurement that uses dual-energy logarithmic intensities.

### 2.1. Dual-Layer Flat-Panel Detector

**Figure 1:**
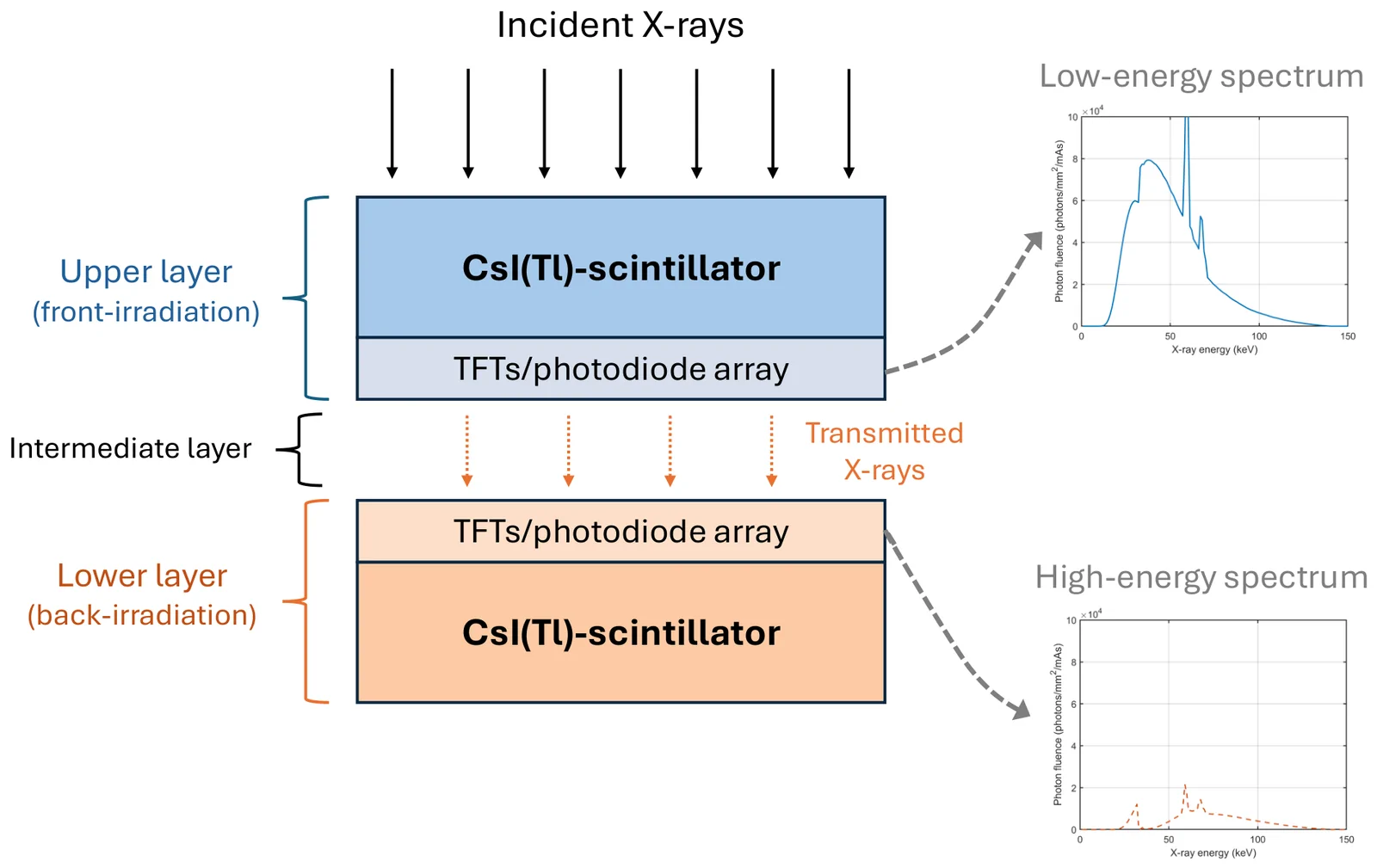
Structure of DFD and the polychromatic spectra. Incident X-rays are first absorbed in the upper scintillator layer, while transmitted photons are subsequently detected by a relatively thick lower scintillator layer in a back-irradiation geometry. A metal filter can be inserted in the intermediate layer to further shape the absorbed spectrum at the lower scintillator layer. This stacked FD structure can increase the photon efficiency.

Several DFD prototypes have been developed by DRTECH Co. Ltd., Gyeonggi, Republic of Korea (www.drtech.com), and their imaging performance has been compared [12,13]. Among these devices, the DFD configuration shown in Figure 1 adopts an inverted lower layer based on a back-irradiation geometry to enhance the modulation transfer function [14,15]. This detector employs an indirect-conversion CsI(Tl)-scintillator, and the lower layer incorporates a relatively thick scintillator to maximize the absorption of photons that are not absorbed in the upper layer and pass through it. With this dual-layer structure, the DFD exhibits a significantly improved detective quantum efficiency (DQE) [13,16], and the total DQE approximately corresponds to the sum of the DQEs of the upper and lower layers [12].

During the stacking of the two FDs, rotational alignment can be precisely adjusted in the manufacturing process; however, subpixel-level misalignment in the horizontal and vertical directions inevitably occurs [8]. In addition, the image acquired in the lower layers is more magnified than that from the upper layer, depending on the source–image distance (SID). Various registration methods have been proposed to correct these alignment errors [17,18]. In contrast, for material decomposition tasks such as BMD measurement, the geometric alignment does not significantly affect material separation, and it is more important that the upper and lower spectra contain sufficient information for BMD measurement.

### 2.2. Spectrum Synthesis in Dual-Energy Imaging

To compare the BMD measurement performance of the dual-shot and single-shot approaches for different scintillator thicknesses, we consider three simulation cases summarized in Table 1. Spectrum synthesis was performed using the SPEKTR 3.0 [10] simulation tool. SPEKTR 3.0 generates X-ray spectra in units of photons/mm^2^/mAs at 100 cm from the source. All spectra were synthesized under the same mAs and source–image distance (SID) conditions (1 mAs, 100 cm).

**Figure 2:**
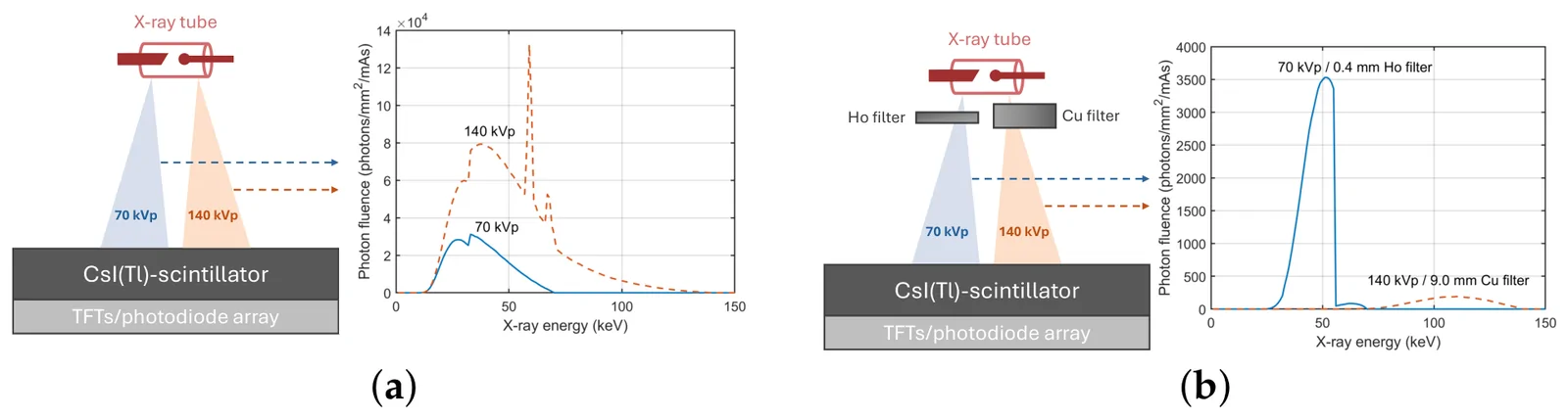
Dual-shot approach in dual-energy imaging and spectrum examples simulated for the FD with a 0.50 mm scintillator layer. (**a**) Spectra obtained at tube voltages of 70 kVp and 140 kVp without additional metal filters. (**b**) Spectra obtained when the 0.40 mm Ho filter and 9.00 mm Cu filter are applied to the low- and high-energy spectra, respectively. The spectra show that spectral overlap is reduced and separation is improved.

Case D represents the FD-based dual-shot approach illustrated in Figure 2, in which a single-layer FD is used to obtain dual-energy spectra by performing two exposures at different X-ray tube voltages in the range of 30–70 kVp, and the high-energy spectrum is synthesized using voltages in the range of 100–140 kVp. The thickness of the scintillator in the FD is fixed at 0.5 mm, and to analyze the impact of the metal filters on BMD measurement performance, a holmium (Ho) K-edge filter with a thickness of 0 or 0.4 mm and a copper (Cu) high-pass filter with a thickness of 0 or 9.0 mm—which are generally considered thickness settings in dual-energy radiography [19,20]—are applied to the low- and high-energy exposures, respectively. Figure 2a shows example low- and high-energy spectra for Case D obtained at tube voltages of 70 kVp and 140 kVp without metal filters. Figure 2b presents the corresponding spectra when a 0.4 mm Ho filter and a 9.0 mm Cu filter are applied in the low- and high-energy beams, respectively, showing that the spectral overlap is substantially reduced and that the filtered configuration yields greater spectral separation than the no-filter case. A thick Cu filter in front of the X-ray tube can substantially reduce beam fluence. While thick Cu filters like 5.0 mm or 9.0 mm are used in industrial applications, thinner Cu filters such as 1.0 mm and 2.0 mm are used in medical applications. In this study, simulations using a thick copper filter were performed to analyze and compare the BMD noise sensitivities when the spectrum is further separated [8].

**Figure 3:**
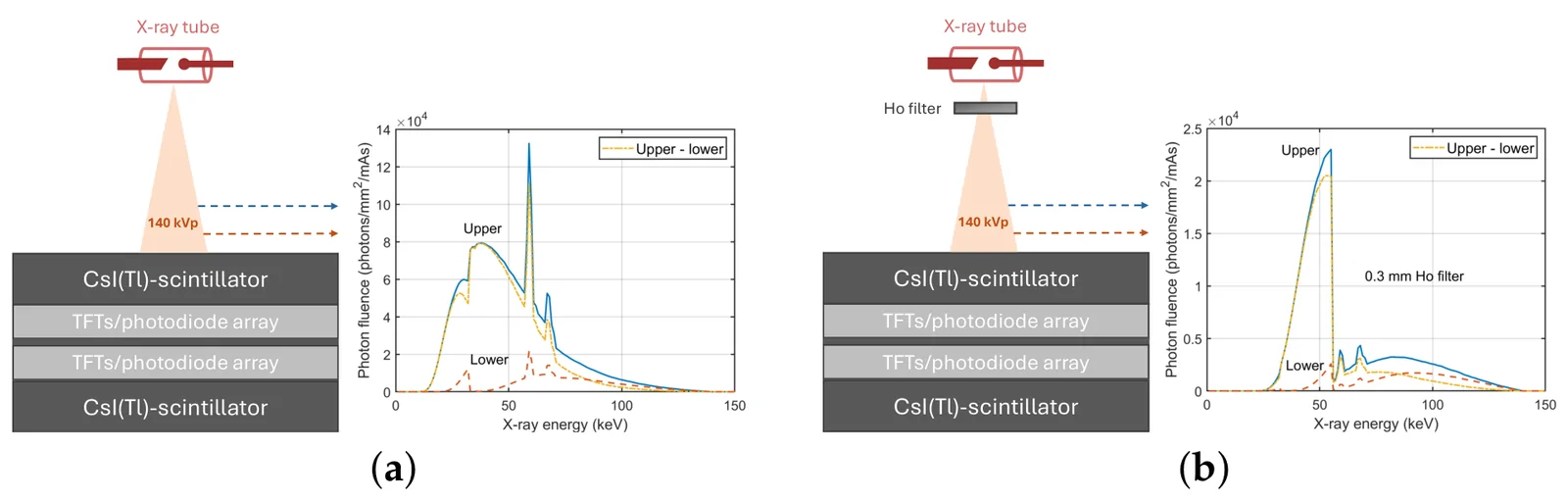
Single-shot approach in dual-energy imaging and spectrum example simulated for the DFD with 0.50 mm upper and lower scintillator layers. (**a**) Upper and lower spectra and the corresponding subtracted spectrum obtained from an exposure at a tube voltage of 140 kVp. (**b**) Upper and lower spectra when a 0.30 mm Ho filter is inserted, where the spectral overlap is partially suppressed.

Case S represents the DFD-based single-shot approach illustrated in Figure 3, in which dual-energy spectra are obtained from a single exposure at a fixed tube voltage of 140 kVp. In this case, the upper and lower spectra correspond to the low- and high-energy spectra, respectively. The thickness of the upper scintillator is varied between 0.20 mm and 0.50 mm, while the thickness of the lower scintillator is fixed at 0.50 mm. To improve spectral separation for the DFD case, the Ho filter can also be employed, and its thickness is varied in a range of 0 to 2.0 mm.

To further mitigate overlap between the two spectra, an intermediate filter could be inserted in the intermediate layer of Figure 1. When a high-pass filter such as Cu is placed in the intermediate layer of the DFD [21,22], it can more effectively reduce the low-energy component of the lower spectrum than beam hardening alone [23]. Such an intermediate filter can slightly improve BMD measurement performance in the single-shot approach [18], but it also degrades the DQE. Therefore, in this paper, we do not use an intermediate filter, in order to maintain high DQE values while focusing on BMD measurement performance.

Subtracting the lower spectrum from the upper spectrum can enhance spectral separation by mathematically removing the high-energy component present in the upper spectrum. We refer to this method as the spectral subtraction and define the corresponding configuration as Case S-sub. This subtracted spectrum replaces the original upper spectrum and is used as low-energy spectrum. Figure 3a illustrates spectrum examples for Case S and Case S-sub without the Ho filter. Compared with the Case D spectra in Figure 2a, Case S exhibits much greater overlap between the two spectra. Figure 3b shows the corresponding spectra for Case S and Case S-sub with a 0.30 mm Ho filter inserted, which partially suppresses the spectral overlap [24].

### 2.3. Bone Mineral Density Measurement

For the simulations, a virtual phantom was constructed with overlapping bone and soft tissue, modeled using aluminum (Al) and acrylic, respectively. Al exhibits similar attenuation characteristics to bone under X-ray irradiation and is widely used in DXA quality-control phantoms, while acrylic serves as a soft-tissue equivalent [25,26]. The BMD of this virtual phantom was synthesized by varying the bone thickness $d_{B}$, to produce BMD values between 0.25 g/cm^2^ and 1.75 g/cm^2^, while the soft tissue thickness $d_{T}$ was randomly varied from 10 cm to 16 cm [27,28]. Using the virtual phantom, 600 samples were generated for each simulation case and were divided into 500 for training and 100 for testing samples. The training samples were used to fit the second-order polynomial estimator, and the test samples were used for noise sensitivity evaluation.

The BMD value is defined as $M_{B}=\rho_{B}d_{B}\;\left(g/{\mathrm{cm}}^{2}\right)$, where $\rho_{B}\approx 2.70\;g/{\mathrm{cm}}^{3}$ is the density of Al. For a photon energy E, the spectrum absorbed in the scintillator layer after passing through the BMD virtual phantom is defined as

(1) $$e^{-\mu_{B}\left(E\right)d_{B}-\mu_{T}\left(E\right)d_{T}}I\left(E\right)F\left(E\right),$$

where $\mu_{B}\left(E\right)$ and $\mu_{T}\left(E\right)$ are the linear attenuation coefficients of bone and soft tissue, respectively. I(E) is the incident spectrum generated by the X-ray tube and directed toward the BMD virtual phantom, and F(E) is the energy-dependent absorption spectrum determined by the detector scintillator. Because the spectrum generated in a real X-ray system is polychromatic, the photon energy E spans a continuous range, and the total intensity at the scintillator layer can be defined as $J:=\int e^{-\mu_{B}\left(E\right)d_{B}-\mu_{T}\left(E\right)d_{T}}I\left(E\right)F\left(E\right)dE$.

Let us denote the logarithms of the integrated intensities by $\psi_{L}:=\ln\left(J_{L}\right)$ and $\psi_{H}:=\ln\left(J_{H}\right)$, which correspond to the logarithmic intensities in the low- and high-energy spectra, respectively. According to the mean value theorem, there exists an energy $E_{L}$ such that

(2) $$\begin{array}{c} \psi_{L}=-\mu_{B}\left(E_{L}\right)d_{B}-\mu_{T}\left(E_{L}\right)d_{T}+\ln\int I\left(E\right)F_{L}\left(E\right)dE \end{array}$$

for the low-energy absorption spectrum $F_{L}$, where the thicknesses $d_{B}$ and $d_{T}$ are fixed. In a similar manner to $\psi_{L}$, we can obtain a relationship for the high-energy logarithmic intensity $\psi_{H}$. From these two relationships on $\psi_{L}$ and $\psi_{H}$, we can derive a relationship between $d_{B}$ and a feature vector $\psi :=(\psi_{L},\psi_{H})$ as

(3) $$\begin{array}{c} d_{B}\left(\psi_{P}\right)=\frac{\Gamma -\psi_{L}+r_{T}\psi_{H}}{\mu_{B}\left(E_{L}\right)-r_{T}\mu_{B}\left(E_{H}\right)}, \end{array}$$

where Γ is a constant derived from the background average intensities, defined as $\Gamma :=\ln\int I_{L}\left(E\right)dF_{L}\left(E\right)-r_{T}\ln\int I_{H}\left(E\right)dF_{H}\left(E\right)$, and $r_{T}$ is an attenuation ratio of the soft tissue, defined as $r_{T}:=\mu_{T}\left(E_{L}\right)/\mu_{T}\left(E_{H}\right)$. Assume that the relationship (3) approximately holds over a narrow range of $d_{B}$ and $d_{T}$. The BMD surface $M_{B}\left(\psi \right)$ can be approximated by a first-order comparametric polynomial of the form

(4) $$M_{B}\left(\psi \right)=\rho_{B}d_{B}\approx f_{1}\left(\psi \right):=a_{0}+a_{1}\psi_{L}+a_{2}\psi_{H},$$

where the coefficients $a_{0}$, $a_{1}$, and $a_{2}$ are estimated by the multiple linear regression using the training samples. Equation (4) defines a hyperplane in the three-dimensional space of $\left(\psi_{L},\psi_{H},M_{B}\right)$. However, because the BMD surface in practical applications is not perfectly planar, a second-order polynomial defined as

(5) $$f_{2}\left(\psi \right):=a_{0}+a_{1}\psi_{L}+a_{2}\psi_{H}+a_{3}\psi_{L}\psi_{H}+a_{4}\psi_{L}^{2}+a_{5}\psi_{H}^{2}$$

can be used for more accurate modeling over a narrow range of $d_{B}$ and $d_{T}$. The BMD value is thus estimated through the second-order polynomial as $M_{B}\left(\psi \right)\approx f_{2}\left(\psi \right)$, which is the polynomial estimator for the BMD measurements in this paper using the dual-energy logarithmic feature ψ. Note that the polynomial coefficients in (5) are re-estimated separately for each combination of tube voltage, metal filter, and scintillator thickness.

### 2.4. Noise Sensitivity Analysis

In this section, to quantitatively compare the noise sensitivities of the dual-shot and single-shot approaches for different scintillator thicknesses, we introduce the absolute condition number and MSE of the polynomial estimator based on multiplicative noise compared to the previous additive noise [8].

The performance of BMD measurement strongly depends on how much input noise is amplified in the estimate—that is, on the noise sensitivity. To compare the noise sensitivities of the simulation cases, we examine the absolute condition numbers of the BMD function and the MSE curve of the BMD estimates. Even though the statistics of pixel intensities follow the Poisson distribution depending on various parameters, average pixel values or average intensities are usually used as the inputs for the BMD measurements. Hence, only the average values of the quantum statistics affect the measurement. Because of instabilities in the X-ray tube current, the average number of emitted photons is not constant but fluctuates substantially, which causes the measured average intensity for a region to vary between acquisitions even for the same object [29,30]. Assuming that this intensity is proportional to the emitted X-rays, the noise can be modeled as being multiplicatively applied to the intensity. Although the approximate shape of the statistical distribution of average intensity, which varies with each image acquisition, could be determined through experimental modeling of X-ray tube instability, a uniform distribution is assumed for the noise sensitivity analysis to simplify the analysis. Similar results can be obtained even when performing the analysis under the assumption of other distributions. Therefore, in this paper, we modeled noise on the basis of a multiplicative noise process, without explicitly modeling the detailed statistics of quantum noise.

As the first method for analyzing noise sensitivity, we observed the absolute condition number of the polynomial estimator. The condition number is a metric that quantifies how strongly small perturbations in the input intensity vector ψ are amplified in the BMD estimate when ψ is input into the polynomial estimator $M_{B}\left(\psi \right)$. For a given ψ, we define the absolute condition number as the Euclidean norm $∥\nabla M_{B}\left(\psi \right)∥$, where $\nabla M_{B}\left(\psi \right)$ is a two-dimensional vector consisting of the partial derivatives with respect to $\psi_{L}$ and $\psi_{H}$. A smaller value of $∥\nabla M_{B}\left(\psi \right)∥$ indicates that the BMD measurement is less sensitive to input noise and is well conditioned.

As the second method, we evaluated noise sensitivity by adding multiplicative noise to the intensity *J*. Independent and identically distributed, uniform random noise was multiplied with $J_{L}$ and $J_{H}$, respectively, and noisy inputs $\psi_{L}$ and $\psi_{H}$ were then generated by applying the logarithmic transformation. These noisy inputs were input into the polynomial estimator to output noisy BMD values. The variance of the multiplicative noise was varied from $10^{-7}$ to $10^{-3}$ and, for each noise variance, the MSE of the noisy BMD values was calculated.

## 3. Simulation Results

In this section, we present the simulation results of BMD measurement for different tube voltages, metal filter thicknesses, and scintillator thicknesses. We first show the noise sensitivity of the dual-shot approach for different X-ray tube voltages and metal filters. We then investigate performance improvements in the single-shot approach by varying the upper scintillator thickness and the K-edge filter thickness, using the dual-shot result as a reference. Finally, by comprehensively analyzing the design configurations of both approaches, we distill their relative characteristics in terms of BMD measurement performance into a set of observable design trends.

### 3.1. Condition Number of the Dual-Shot Approach

**Figure 4:**
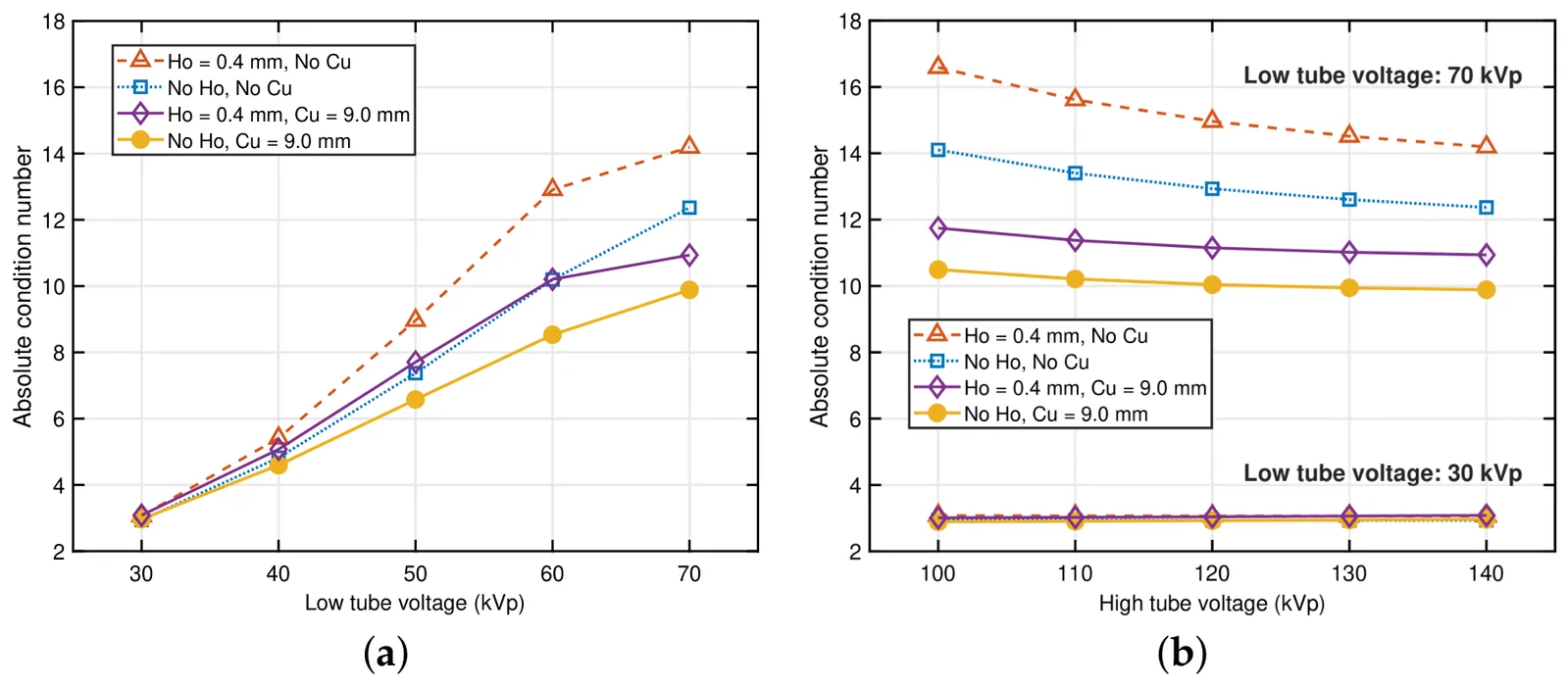
Absolute condition numbers obtained from the second-order polynomial estimator under different metal filter combinations. (**a**) Sweep over low tube voltage with the high tube voltage fixed at 140 kVp. (**b**) Sweep over high tube voltage with the low tube voltage fixed at 30 kVp or 70 kVp. In both cases, the configuration without Ho and with Cu exhibits the lowest absolute condition numbers.

We first introduce the simulation results for Case D. Figure 4a shows the absolute condition number curves obtained by sweeping the low tube voltage while keeping the high tube voltage fixed at 140 kVp for different metal filter combinations of Ho and Cu filters. As the low tube voltage decreases from 70 kVp to 30 kVp, the absolute condition numbers drop markedly. At 70 kVp, adding the Cu filter provides a noticeable improvement in performance (“No Ho, Cu = 9.0 mm”), whereas the Ho filter slightly worsens the performance (“Ho = 0.4 mm, Cu = 9.0 mm”). When the low tube voltage is already reduced to 30 kVp, however, the metal filter configurations have only a minor influence. Figure 4b plots the absolute condition number as a function of the high tube voltage, with the low tube voltage fixed at either 30 kVp or 70 kVp. Across the entire high tube voltage range, the curves for 30 kVp are consistently lower than those for 70 kVp, and variations in the high tube voltage itself have only a minor impact on the noise sensitivity. These results indicate that, in the dual-shot approach, decreasing the low tube voltage is the primary and most effective design strategy, whereas tuning the high tube voltage or metal filters offers only limited additional benefit.

### 3.2. Condition Number of the Single-Shot Approach

**Figure 5:**
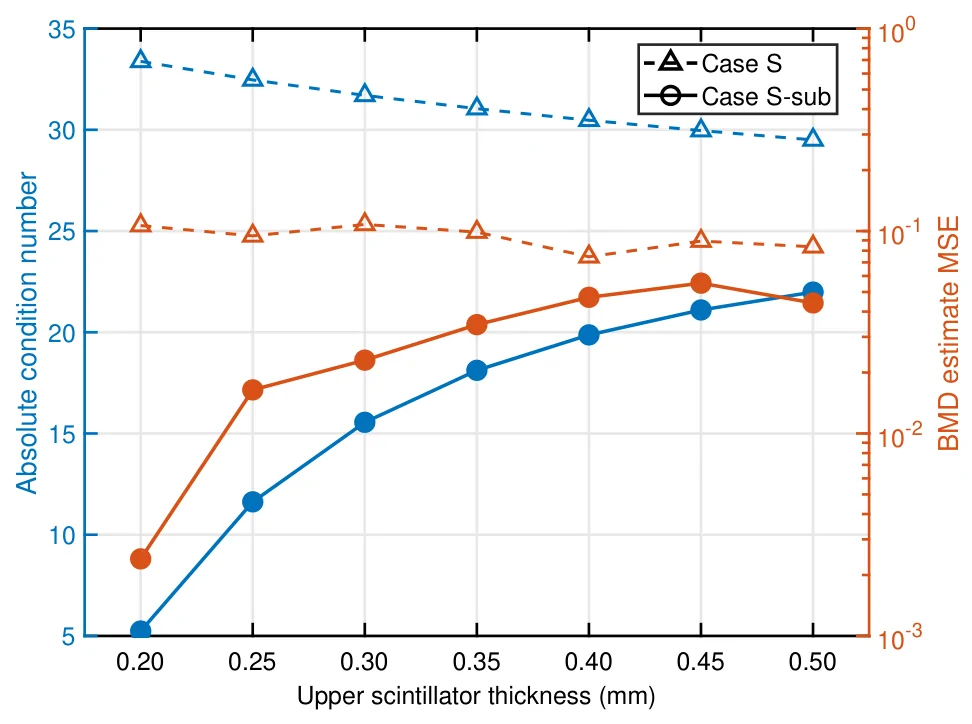
Absolute condition number and BMD estimate MSE with respect to the upper scintillator thickness for Case S and Case S-sub without the Ho filter. For Case S-sub, the best noise sensitivity performance is achieved when the upper scintillator is designed to be thin.

We next present the simulation results for Case S and Case S-sub. Figure 5 shows the absolute condition number and MSE curves of Case S and Case S-sub with respect to the upper scintillator thickness in the absence of the Ho filter. For all scintillator thicknesses, Case S-sub with the spectral subtraction yields consistently lower condition numbers and MSE values than those of Case S. Note that, as the upper scintillator becomes thinner, the noise sensitivity of Case S-sub improves, whereas that of Case S tends to slightly degrade.

**Figure 6:**
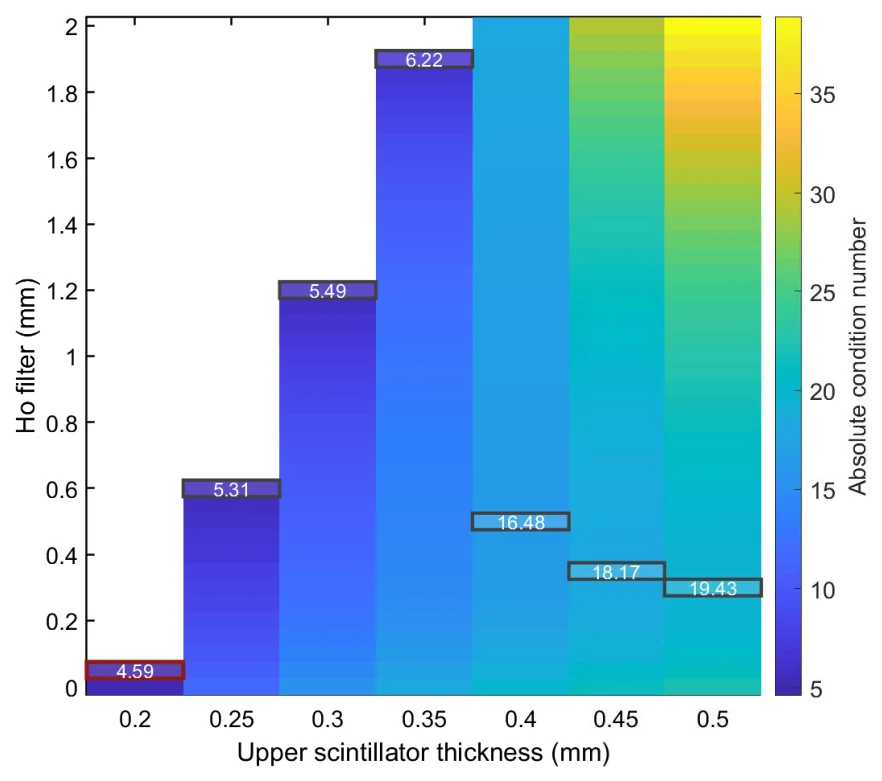
Heatmap of the absolute condition number for Case S-sub over the design space of upper scintillator thickness and Ho filter thickness. The numerical labels mark, for each upper scintillator thickness, the minimum absolute condition number obtained at a given Ho filter thickness, and the globally lowest value occurs at the thinnest upper scintillator layer.

Figure 6 visualizes the absolute condition number distribution of Case S-sub as a heatmap obtained by sweeping the upper scintillator thickness and the Ho filter thickness over a two-dimensional grid with a step size of 0.05 mm in each direction. Optimal condition numbers are clearly concentrated in the region of thinner scintillators, which indicates that, in the single-shot approach, BMD measurement performance is primarily determined by the upper scintillator thickness. An interesting feature of the heatmap is that, for thin scintillator settings (0.20–0.35 mm), the optimal region shifts toward larger Ho thickness, while no such shift is seen for thick scintillator settings (0.40–0.50 mm). In addition, the blank region in the heatmap represents combinations of upper scintillator and Ho filter thicknesses for which the condition number is not defined.

**Figure 7:**
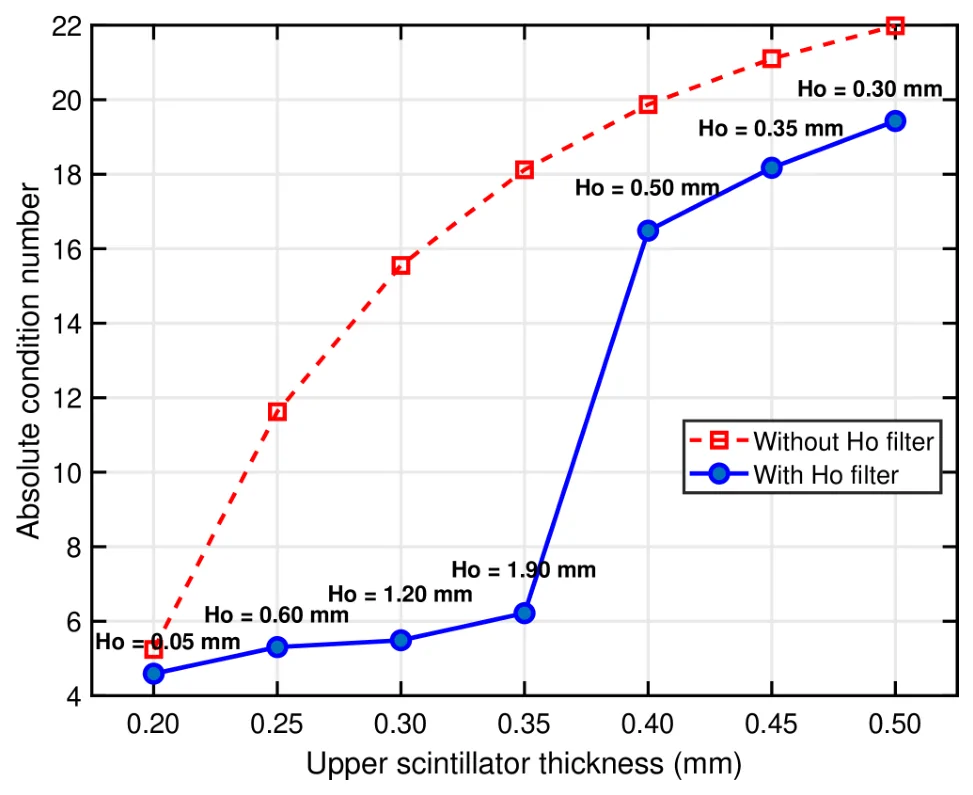
Absolute condition number of Case S-sub with respect to the upper scintillator thickness, comparing curves with and without the Ho filter. For the curve with the Ho filter, each point is annotated with the optimal Ho thickness selected at that upper scintillator thickness. Consistent with Figure 6, the trend in the optimal Ho thickness changes abruptly in the thick scintillator settings.

In Figure 7, the absolute condition number curves of Case S-sub are compared for different scintillator thicknesses, between the case without the Ho filter and the case with the optimal Ho filter thickness for each scintillator thickness. At a scintillator thickness of 0.20 mm, which yields the lowest condition number within the examined thickness range of 0.20 mm to 0.50 mm, the difference between using no Ho filter and using the optimal Ho filter thickness is negligible. This indicates that, in the single-shot approach, if the upper scintillator is designed to be sufficiently thin, the Ho filter can effectively be an optional component.

### 3.3. Comparison of the Mean Square Errors

**Figure 8:**
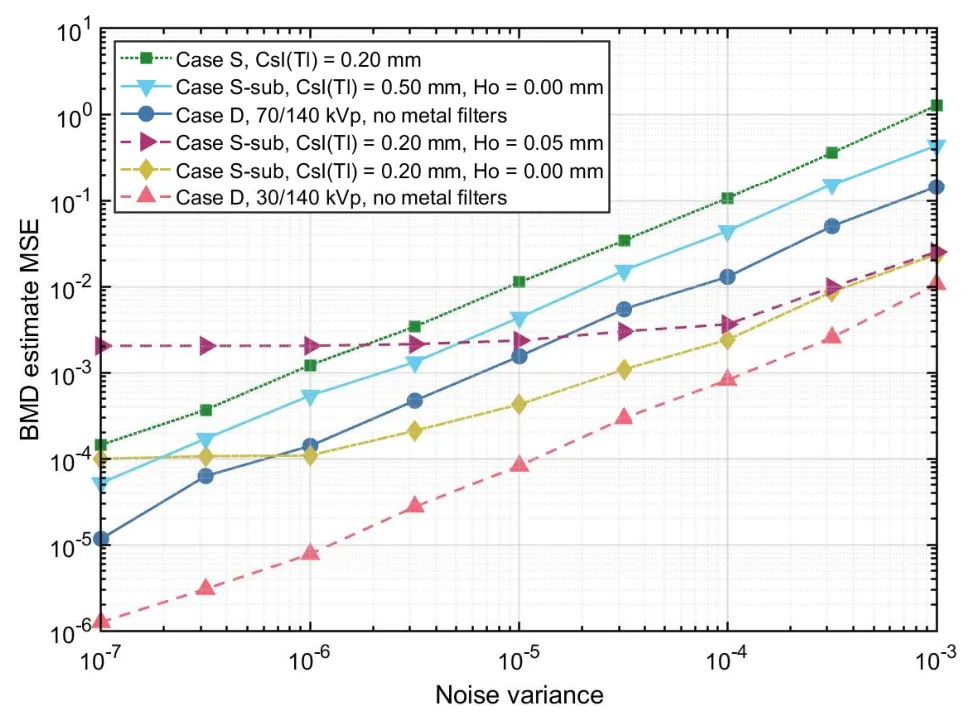
BMD estimate MSE curves with respect to multiplicative noise variance for representative dual-shot and single-shot approach simulations.

Figure 8 summarizes the BMD estimate MSE curves with respect to the multiplicative noise variance for the simulation cases, Case D, Case S, and Case S-sub. As the noise variance increases, the MSE values also increase. In the dual-shot approach based on FD, a low tube voltage of 30 kVp (“Case D, 30/140 kVp, no metal filters”) shows a better MSE curve than the case of 70 kVp (“Case D, 70/140 kVp, no metal filters”). In the single-shot approach, Case S-sub (with the spectral subtraction scheme) consistently outperforms Case S across all design configurations. Within the simulations of Case S-sub, reducing the upper scintillator thickness yields the most pronounced performance gains, and when the upper scintillator is sufficiently thin, adding the Ho filter provides no meaningful improvement in terms of the noise sensitivity. Comparing the optimized Case S-sub (“Case S-sub, CsI(Tl) = 0.20 mm, Ho = 0.00 mm”) with the optimized Case D (“Case D, 30/140 kVp, no metal filter”) shows that their MSE curves are similar, indicating that the single-shot approach can achieve BMD measurement accuracy comparable to that of the dual-shot approach.

## 4. Discussion

In this section, we discuss how different hardware settings affect the performance of the BMD measurement methods. We first examine the influence of the tube voltage in the dual-shot approach. We then consider the effects of the upper scintillator thickness in the single-shot approach.

**Figure 9:**
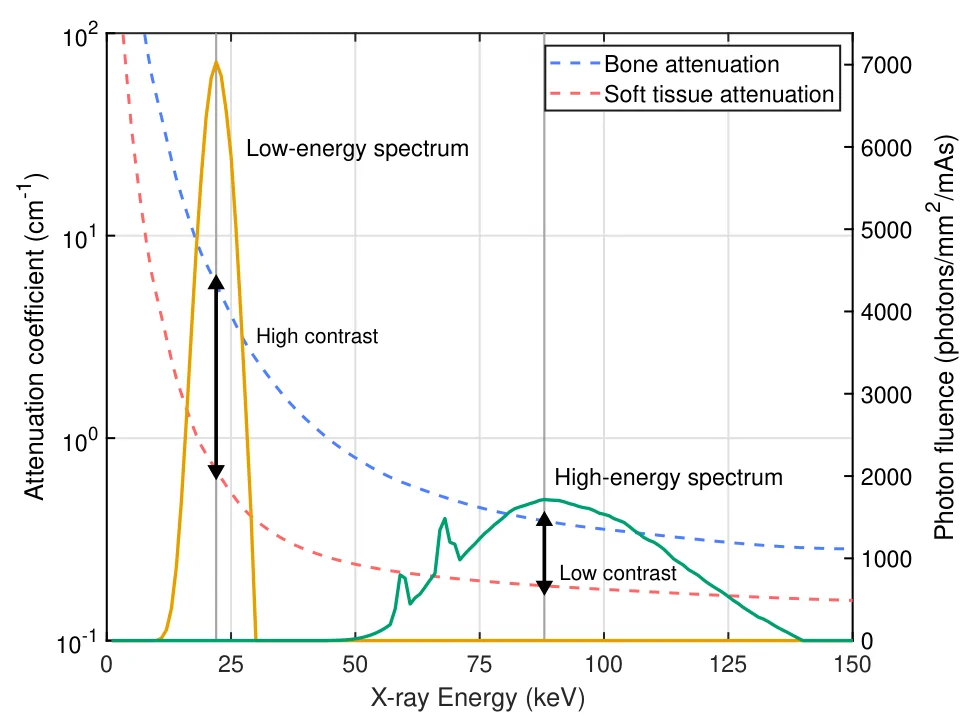
Linear attenuation coefficients of bone and soft-tissue together with example with absorbed spectra for low- and high-energy beams. The attenuation coefficients were obtained from SPEKTR 3.0 (NIST XCom-based). The low-energy spectrum samples an energy range where the difference in linear attenuation coefficients between the bone and soft tissue is larger than the high-energy spectrum case.

We consider the equivalent energies $E_{L}$ and $E_{H}$ and the bone-tissue attenuation ratios to interpret the physical relationship between dual-energy spectral design and noise sensitivity in measuring BMD values. Even though the equivalent energies are defined for the polychromatic spectra, these energies can be considered equivalent to the low and high energies of monochromatic spectra to facilitate interpretation of their effects on BMD measurement performance. Using these equivalent energies and from (3), the absolute condition number can have an approximation as

(6) $$\begin{array}{c} ∥\nabla M_{B}\left(\psi \right)∥\approx \frac{\rho_{B}\sqrt{1/\mu_{T}^{2}\left(E_{L}\right)+1/\mu_{T}^{2}\left(E_{H}\right)}}{R\left(E_{L}\right)-R\left(E_{H}\right)}, \end{array}$$

where the bone-tissue attenuation ratio is defined as $R\left(E\right):=\mu_{B}\left(E\right)/\mu_{T}\left(E\right)$. For a fixed high tube voltage or $E_{H}$, decreasing the low tube voltage or $E_{L}$ can increase both $\mu_{T}\left(E_{L}\right)$ and the ratio $R(E_{L})$, as demonstrated in Figure 9, and can eventually decrease the absolute condition number from (6). Based on this analysis, we observe the equivalent energies and bone-tissue attenuation ratios of the simulation cases to discuss the simulation results on the tube voltage and scintillator thickness.

### 4.1. Tube Voltages in the Dual-Shot Approach

**Figure 10:**
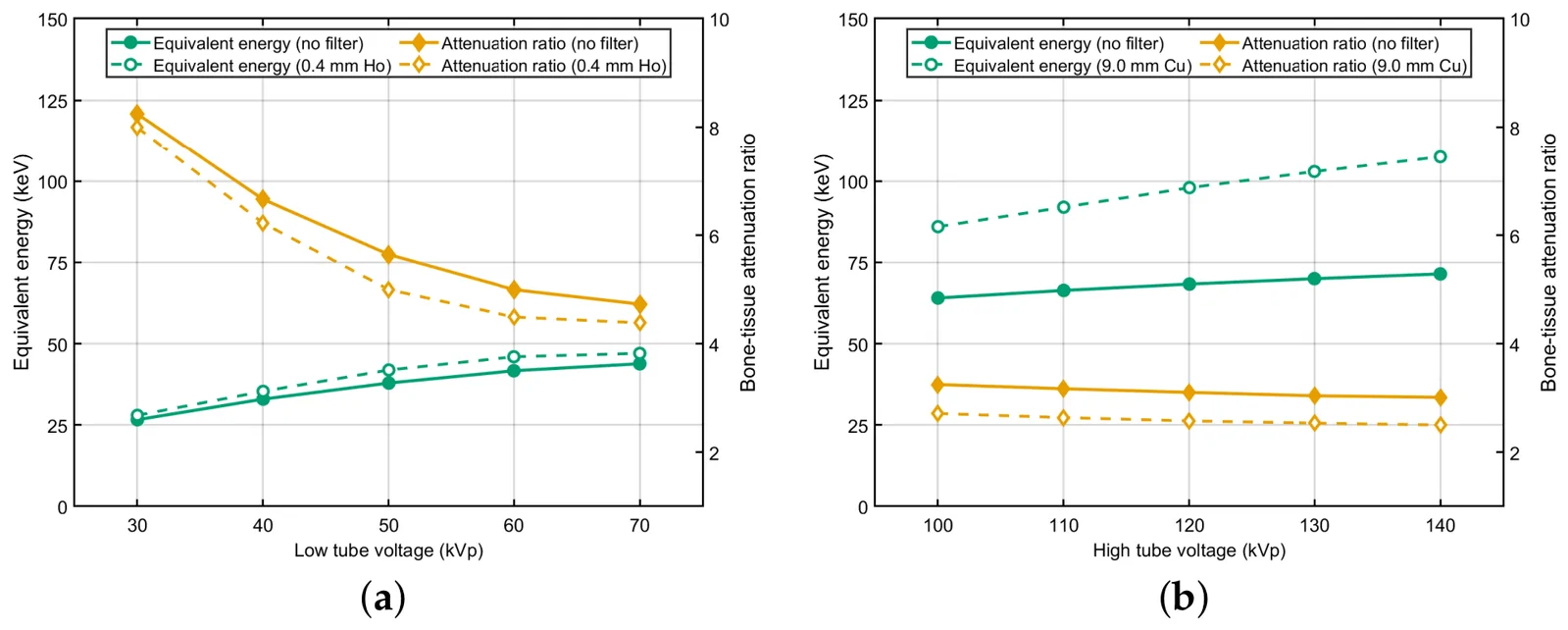
Equivalent energies and bone-tissue attenuation ratios for Case D, where the Ho and Cu filters are also considered. (**a**) Low equivalent energy $E_{L}$ and low ratio $R(E_{L})$ with respect to the low tube voltage. (**b**) High equivalent energy $E_{H}$ and high ratio $R(E_{H})$ with respect to the high tube voltage.

In Figure 10, the equivalent energies and attenuation ratios with respect to the tube voltage are illustrated for Case D. We can observe from Figure 10a that, as the low tube voltage decreases for a fixed high tube voltage of 140 kVp, the low equivalent energy $E_{L}$ also decreases and the corresponding ratio $R(E_{L})$ increases consequently. Hence, from (6), the condition number is improved. On the other hand, using the Ho filter for the exposure of the low tube voltage hardly reduces $E_{L}$ and even slightly increases it compared to the no-filter case. The K-absorption edge of Ho (≈55.6 keV) [31] lies well above the mean energies of the low-energy spectrum in a tube voltage range of 30–70 kVp exposures. Consequently, the Ho filter does not substantially improve spectral separation; instead, it hardens the low-energy spectrum, slightly increasing $E_{L}$. In contrast, Figure 10b indicates that, despite variations in the high tube voltage, the high equivalent energy $E_{H}$ and the corresponding ratio $R(E_{H})$ remain almost unchanged. Nevertheless, using the Cu filter for the exposure of the high tube voltage can raise $E_{H}$, which can contribute to some improvement in material separation by widening the energy gap between the two spectra. However, when $E_{L}$ is already sufficiently low, the Cu filter no longer produces a meaningful improvement. Hence, we notice that decreasing the tube voltage for the low-energy spectrum can effectively improve the material decomposition performance, rather than employing metal filters or adjusting the high tube voltage in the dual-shot approach.

### 4.2. Upper Scintillator Thickness in the Single-Shot Approach

**Figure 11:**
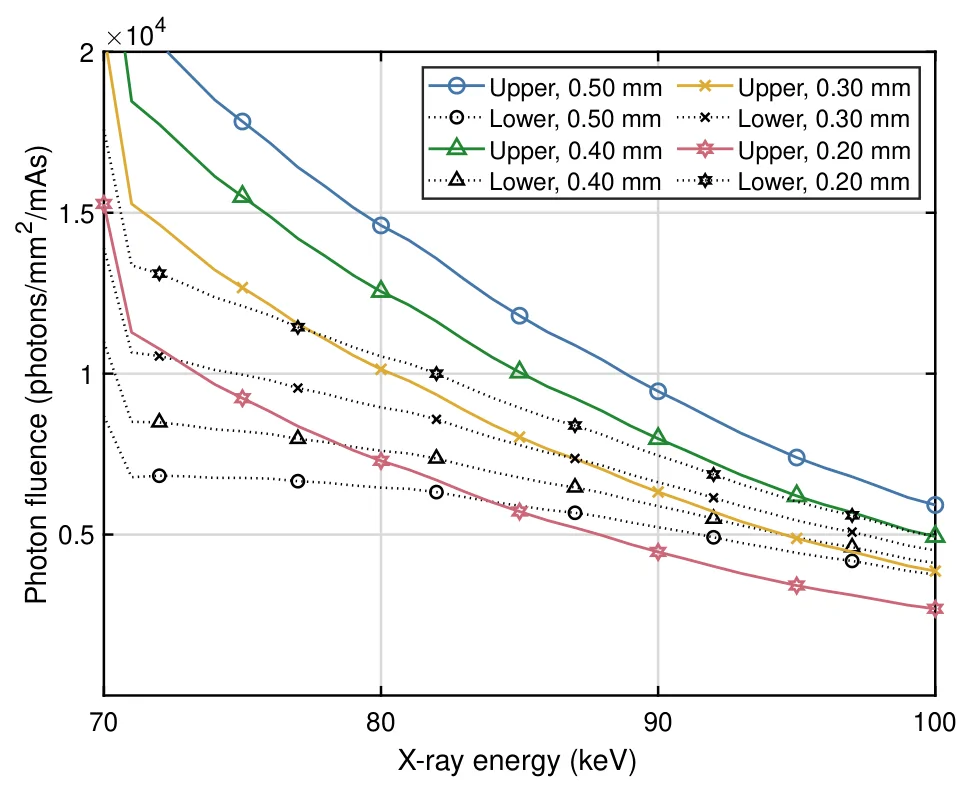
Simulated absorbed spectra for the upper and lower scintillator layers at different upper scintillator thicknesses, shown only over a clipped high-energy range, with the lower scintillator thickness fixed at 0.5 mm. For an upper thickness of 0.50 mm, the upper spectrum exhibits a higher fluence than the corresponding lower spectrum across the high-energy range, whereas at 0.20 mm this ordering is reversed, indicating the scintillator thickness-dependent change in the relative response scales of the two layers.

For a relatively high-energy region of 70–100 keV, Figure 11 shows photon fluence curves simulated for the DFD. As the upper scintillator becomes thinner, the response scale of the upper-layer spectrum progressively decreases, whereas that of the lower-layer spectrum shows the opposite tendency. For the low-energy intensities, this property generates a subtraction spectrum in which, as the upper scintillator layer becomes thinner, it is more strongly weighted toward the low-energy region.

**Figure 12:**
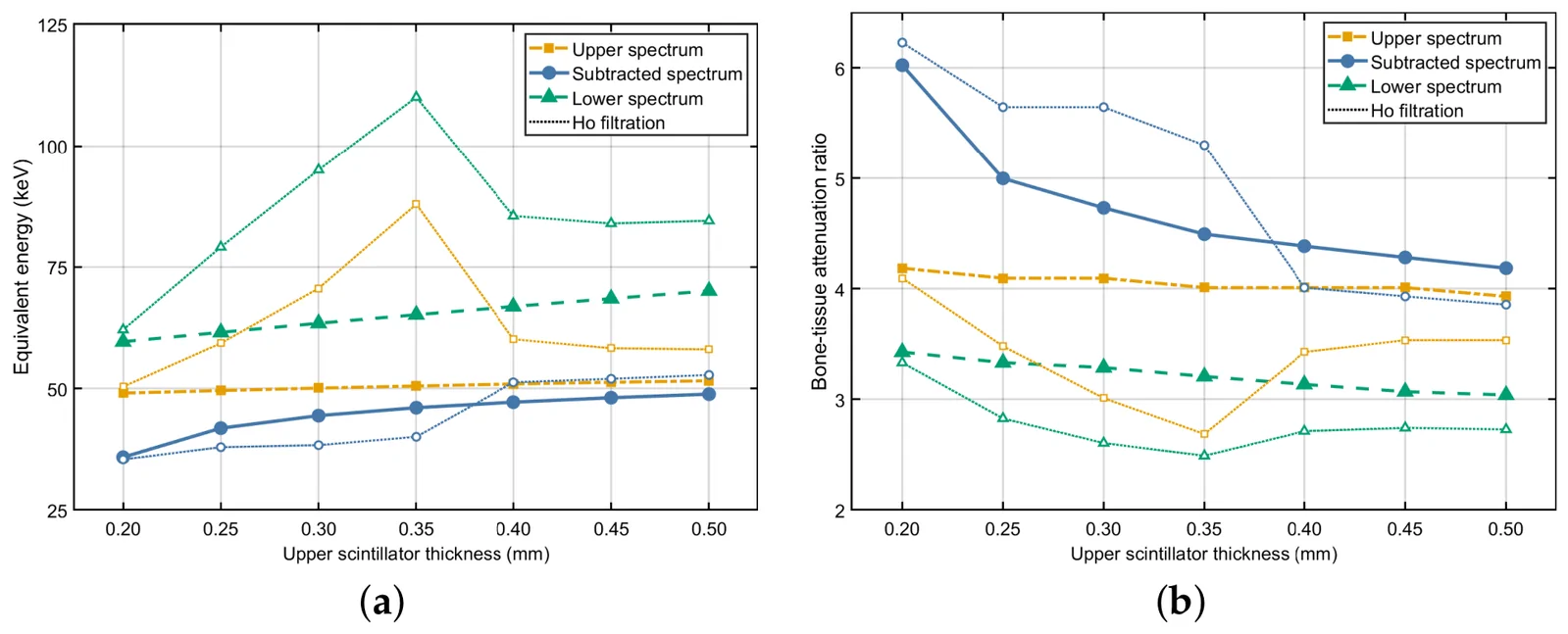
Equivalent energies and bone-tissue attenuation ratios for Case S and Case S-sub, where the Ho filter is also considered, with the optimal Ho thickness selected at each upper scintillator thickness. (**a**) Equivalent energy curves with respect to the upper scintillator thickness. (**b**) Bone-tissue attenuation ratio curves with respect to the upper scintillator thickness.

For Case S and Case S-sub, Figure 12 illustrates the equivalent energies and attenuation ratios of spectra with respect to the upper scintillator thickness. As the upper scintillator becomes thinner, $E_{L}$ decreases and the corresponding $R(E_{L})$ increases. From (6), these trends confirm that thin scintillator settings decrease the absolute condition number and, thus, improve the material decomposition performance.

Furthermore, the curves of Case S-sub exhibit a more pronounced decrease in $E_{L}$ and increase in $R(E_{L})$ compared to those of Case S, demonstrating that the Case S-sub-based BMD measurement method can provide better noise sensitivity than the Case S method in a single-shot approach through spectral subtraction. When the Ho filter is employed, $E_{L}$ in Case S tends to increase, leading to a concomitant decrease in $R(E_{L})$, whereas in Case S-sub it slightly decreases under thin scintillator settings and, thus, yields improved performance.

The K-edge filter sharply absorbs photons around a specific energy, allowing a single X-ray spectrum to be split into distinct low- and high-energy peaks. Such filters can enhance spectral separation between two energy spectra in a DFD. When the Ho filter of appropriate thickness is inserted, the low- and high-energy peaks become more clearly separated, and applying the spectral subtraction in this state maximizes the spectral separation. Accordingly, under thin scintillator settings, a properly thick Ho filter provides an effective design configuration. However, when the upper scintillator is thin, fewer X-ray photons contribute to the upper spectrum, and its overall response scale becomes smaller. In this regime, an excessively thick Ho filter can invert the relative scales of the upper and lower spectra, leading to negative values, which are reflected by the blank region in Figure 6.

Under thick scintillator settings, the trend in the optimal Ho filter thickness changes. When the upper scintillator is thick, the larger number of absorbed X-ray photons keeps the response scale of the upper spectrum higher than that of the lower spectrum even in the high-energy region of Figure 11. As a result, the spectral subtraction provides only limited improvement in spectral separation. Increasing the Ho filter thickness further in the same regime no longer enhances separation and instead raises $E_{L}$. Hence, when the upper scintillator is thick, a thick Ho filter is not always advantageous, and this characteristic is consistent with the simulation results in Figure 6. Here, optimal performance is achieved when the Ho filter thickness is in the range of 0.30 mm to 0.50 mm.

### 4.3. Practical Limitations

When developing a BMD measurement system using indirect-conversion FDs, issues such as the nonlinearity in detector responses and X-ray beam scattering should be considered. The output photocurrent becomes a nonlinear function of the incident radiation intensity due to the photodiode and scintillator [32]. This nonlinearity reduces the collection efficiency of generated charge carriers and causes the output signal to be disproportionately lower than the incident X-ray intensity [33]. In cone-beam-based imaging systems, scattered radiation is one of the major causes of degradation in quantitative accuracy [34]. X-ray scatter distorts the measured pixel intensities and can reduce detection performance [35,36] in low-contrast regions, particularly in applications such as BMD measurements, where the difference in linear attenuation between materials is relatively small [37]. To mitigate scattering distortion, anti-scatter grids [38] and scatter correction techniques have been proposed [39,40]. For practical experiments using physical step Al and acrylic phantoms, or BMD phantoms, these problems of the detector nonlinearity and beam scattering should be carefully considered to implement the simulation results introduced in this paper.

## 5. Conclusions

In this paper, we conducted simulations on optimizing the scintillator thickness in measuring BMD values based on a DFD and demonstrated that this DFD-based single-shot approach can achieve similar noise sensitivities and simulated MSE values compared to those of the FD-based dual-shot approach. We also optimized and analyzed the performance, additionally exploring the effects of tube voltage and metal filter settings. The analyses in this paper, based on extensive simulations, can be summarized as follows:

- In the dual-shot approach based on an FD, decreasing the low tube voltage provides the most substantial performance improvement in terms of the noise sensitivity, whereas combining metal filters for separating dual-energy spectra yields only limited benefits.
- In the single-shot approach based on a DFD, applying spectral subtraction enhances both spectral and material separation, and when the upper scintillator is designed to be sufficiently thin within the investigated range (0.20 mm – 0.50 mm), low noise sensitivity can be achieved even without the K-edge filter.

These findings suggest that the single-shot BMD measurement using a DFD has the potential to serve as an alternative to conventional dual-shot methods and may potentially be extended to high-efficiency opportunistic screening applications in the future. For practical clinical applications, future work will employ various physical phantoms and conduct more realistic comparative experiments that account for detector nonlinearity and scatter distortion.

## Figures and Tables

**Table 1:** Various simulation cases for dual-shot and single-shot approaches, including detector configuration, tube voltage, and filter settings, where a 1.6 mm inherent Al filter is considered.

Case	X-Ray Tube Voltage	Metal Filter		Scintillator CsI(Tl)	Spectral Subtraction
		Ho	Cu		
D	30–70/100–140	0, 0.4	0, 9.0	0.5	Not used
S	140	0–2.0	None	0.2–0.5	Not used
S-sub					Used

The units of X-ray tube voltage and all thicknesses are kilovolt peak (kVp) and millimeters (mm), respectively.

## Data Availability

The original contributions presented in this study are included in the article; further inquiries can be directed to the corresponding author.

## Abbreviations

The following abbreviations are used in this manuscript:

BMD	Bone mineral density
DFD	Dual-layer flat-panel detector
DQE	Detective quantum efficiency
DXA	Dual-energy X-ray absorptiometry
FD	Flat-panel detector
SID	Source–image distance
